# Microbes and pathogens associated with shrimps - implications and review of possible control strategies

**DOI:** 10.3389/fmars.2024.1397708

**Published:** 2024-10-06

**Authors:** Subha Bhassu, Maryam Shama, Suma Tiruvayipati, Tze Chiew Christie Soo, Niyaz Ahmed, Khatijah Yusoff

**Affiliations:** 1 Animal Genetics and Genome Evolutionary Lab (AGAGEL), Division of Genetics and Molecular Biology, Institute of Biological Sciences, Faculty of Science, University of Malaya, Kuala Lumpur, Malaysia; 2 Centre for Research in Biotechnology for Agriculture (CEBAR), University of Malaya, Kuala Lumpur, Malaysia; 3 Malaysian Genome Vaccine Institute, National Institute Biotechnology Malaysia, Bangi, Selangor, Malaysia; 4 Infectious Diseases Translational Research Programme, Department of Medicine, Yong Loo Lin School of Medicine, National University of Singapore, Singapore, Singapore; 5 Department of Biotechnology and Bioinformatics, University of Hyderabad, Hyderabad, Telangana, India

**Keywords:** shrimp, pathogens, diseases, aquaculture, biosecurity

## Abstract

Shrimp aquaculture has been growing rapidly over the last three decades. However, high-density aquaculture together with environmental degradation has led to increased incidence of shrimp infections. Thus, devising and implementing effective strategies to predict, diagnose and control the spread of infections of shrimps are crucial, also to ensure biosecurity and sustainability of the food industry. With the recent advancements in biotechnology, more attention has been given to develop novel promising therapeutic tools with potential to prevent disease occurrence and better manage shrimp health. Furthermore, owing to the advent of the next-generation sequencing (NGS) platforms, it has become possible to analyze the genetic basis of susceptibility or resistance of different stocks of shrimps to infections and how sustainable aquaculture could be made free of shrimp diseases.

## 1 Introduction to shrimp farming and disease management

Shrimp farming has been identified as one of the most profitable aquaculture sectors in the Asia-Pacific region (De Silva et al., 2007). Aquaculture production of shrimp has been increasing globally, dominated by Southeast Asia, China, India, and America (Anderson et al., 2019). Shrimps belonging to the *Penaeidae* family are recognized as a valuable economic resource in crustacean aquaculture sector. Among all farmed shrimps, the black tiger shrimp (*Penaeus monodon*) and the pacific white shrimp (*Litopenaeus vannamei*) contribute to more than 90-95% of the world production. Initially, *P. monodon* was identified as the dominant species among cultured shrimps. However, owing to factors such as lower levels of protein requirements and disease resistance, *L. vannamei* surpassed *P. monodon*, and now attributes to more than 70% of crustacean aquaculture production (Flegel et al., 2008; Arulmoorthy et al., 2020).

Over the past few decades, there has been a rapid growth in penaeid shrimp aquaculture sector. This over-intensification, along with environmental degradation and the introduction of new varieties in the tropics and subtropics, has resulted in an increased occurrence of emerging shrimp diseases (Walker and Mohan, 2009; Thitamadee et al., 2016; Xiong et al., 2016). The production of shrimp is impaired by diseases primarily caused by various microbial pathogens such as viruses, bacteria, fungi, and protozoa. The World Animal Health Organization (OIE: Office International des Epizooties) recognizes certain diseases as the most significant, which were included in their list for penaeid shrimp diseases (Lightner et al., 2012).

Since the late 1980s, unknown diseases have emerged and spread in shrimp aquaculture farms across the globe, causing staggering economic losses in some countries. For instance, Taiwan faced a loss of US$3 billion of farmed shrimp in 1994, and US$0.5 billion of *P. monodon* cultures between 1987 and 1988 (Lundin, 1995). White spot syndrome virus (WSSV) was discovered in Taiwan in the early 1990s, and it quickly spread to major shrimp aquaculture farms in Asian countries such as Japan. WSSV caused high mortality rates, leading to an economic loss of US$6 billion (Afsharnasab et al., 2014). In addition to WSSV, other viral pathogens have also caused epidemics in different regions. While Taiwan encountered monodon baculovirus (MBV) epidemic during the mid-1980s, shrimp farms in America were impacted by infectious hypodermal and hematopoietic necrosis virus (IHHNV) and taura syndrome virus (TSV) in 1981 and 1992, respectively. Simultaneously, in 1990, yellow head virus (YHV) first emerged in cultivated shrimps in Thailand (Walker and Mohan, 2009).

Other significant pathogenic diseases include necrotizing hepatopancreatic bacterium (NHPB) disease (Cuéllar-Anjel et al., 2018) and vibriosis (Chandrakala and Priya, 2017) caused by bacteria. Moreover, there are some other fungal and protozoan parasitic diseases that can infect shrimps, as well. Owing to such diseases, mostly caused by viruses, it was projected that since 1994, the worldwide aquaculture sector has incurred an annual loss of US$3 billion (Lundin, 1995; Lightner, 1999). Although there has been a noticeable recovery over the last few years, the growth and profitability of this sector remains impacted by infectious diseases caused by various microbial pathogens.

Penaeid shrimps, unlike vertebrates, lack adaptive immune system and separate lymphatic systems to protect themselves against invading pathogens; thus, increasing the risk of mortality within a few days of infection. This also makes it challenging to develop vaccines against various pathogens (Johnson et al., 2008; Aguirre-Guzman et al., 2009). Moreover, some pathogens infect shrimps at different life stages, even larvae, that has little or no innate immune response (Lightner et al., 2012). Therefore, the enhancement of biosecurity at shrimp farms, development of rapid diagnostic methods and disease prevention strategies are crucial. This review should serve as a compendium of the major shrimp diseases caused by pathogens, the significance of shrimp gut microbiota, and its correlation with the emergence and occurrence of diseases. Furthermore, in addition to the available pathogen detection, diagnostic, and control strategies, novel technologies for improved detection methods and promising therapeutic tools for shrimp diseases are also reviewed in this paper.

## 2 Pathogen diversity in shrimp aquaculture

### 2.1 Microbial diseases of penaeid shrimps

Microbial pathogens that cause diseases in shrimps belong to various types of viruses, bacteria, fungi, and protozoan parasites. Among these, certain diseases that cause major economic losses, are recognized by OIE as most significant (Table 1).

#### 2.1.1 Viral diseases

##### 2.1.1.1 Taura syndrome (TS)

Taura syndrome virus (TSV) which belongs to the family *Dicistroviridae* is the causative agent of TS. With a diameter of 32 nm, the virion is a naked (without an envelope) icosahedron. TSV’s genetic material consists of 10,205 nucleotides of positive-sense, single-stranded RNA (+ssRNA). TSV’s genome has two open reading frames (ORFs): ORF 1, which codes for non-structural proteins, and ORF 2, which contains TSV structural protein sequences such as capsid proteins (Mari et al., 2002). Studies conducted on the TSV using molecular tools indicated that a single virus strain was behind the first TSV pandemic in American (Ecuador) aquaculture farms from 1991-1992. However, when cDNA sequences of TSV capsid protein 2 (CP2) were compared (Tang and Lightner, 2005), four different genetic variants of TSV were discovered (Wertheim et al., 2009). TS has caused a huge loss to the global shrimp aquaculture industry and has been reported in the Middle East, America, and Asia. The spread of TS was mainly due to the transfer of live broodstocks across regional and international borders (Walker and Mohan, 2009; Lightner, 2011).

With a cumulative death rate of 40-90%, *L. vannamei* is one of the most vulnerable species to TSV. TS has been reported in this species at post-larvae (PL), juvenile, and adult stages of its life cycle (Ochoa et al., 2020). This virus is also known to infect other varieties of shrimps such as *Penaeus stylirostris* and *Penaeus setiferus*. In addition, experiments on PL and juveniles of *Penaeus japonicus*, *P. monodon*, *Penaeus duorarum*, *Fenneropenaeus chinensis*, *Penaeus aztecus* and *Penaeus schmitti* were also reported to be prone to TSV. The viral replication of TSV is in the cytoplasm of the host cell (Ganjoor, 2015). TS occurs in shrimps in three phases. In the peracute/acute phase, the shrimps are more likely to die, and is characterized by the showing of tail fan, pale reddish color in pleopods, softening of coats and hollow intestines. Individuals that survive this phase will go through the regeneration process, which begins with multifocal melanoid lesions. In the chronic infectious phase, the shrimp remains persistently infected with subclinical infections. The TSV is spread to susceptible shrimps by contaminated water and horizontal transmission via cannibalism of diseased, moribund or dead shrimps. It is also hypothesized that TSV can be vertically transmitted, although this is yet to be experimentally validated. Furthermore, the aquatic insect, water boatman *Trichocorixa reticulata* (Guerin-Meneville, 1857), has been identified as a TSV vector (Dhar et al., 2004). Since the mid-1990s, various research and commercial breeding programs have employed TSV specific pathogen resistance (SPR^1^) selective breeding to combat TSV disease, significantly decreasing its incidence (Sookruksawong et al., 2013). Notably, between 1999 and 2004, there were no TSV outbreaks reported in Colombian shrimp farms, demonstrating the effectiveness of a TSV-resistant breeding program where 100% of the raised shrimp were TSV-SPR^1^.

##### 2.1.1.2 White spot disease (WSD)

WSD is a serious disorder caused by WSSV that causes rapid death, most notably in juvenile shrimp. WSSV is an enveloped virus of the genus *Whispovirus* with double-stranded DNA (dsDNA) and a genome size of 290 to 305 Kbp on average. The size of the genome of isolates from various geographical regions varies, indicating genetic instability that might lead to alterations in virulence (Ganjoor, 2015).

WSSV is widely regarded as one of the most serious threats to the shrimp aquaculture sector (Flegel and Alday-Sanz, 1998). Between 1990 and 1994, the first WSD outbreak was observed in Taiwan and Japan, in *P. japonicus* (Zhu et al., 2019). In 1999, WSSV was discovered in the United States and Latin America, inflicting massive losses in *L. vannamei* and *P. stylirostris* aquaculture. Subsequently, infections were discovered in *P. indicus*, *P. setiferus*, *F. chinensis*, *P. merguiensis*, and *P. monodon*, as well. The geographical spread of WSSV became a severe threat to shrimp farming in Asia and America. This restricted the import requirements for shrimp broodstocks in different countries with a ban on animal imports from regions with viral infection (Sangamaheswaran and Jeyaseelan, 2001; Citarasu et al., 2006; Zhang et al., 2006).

Significant signs of acute WSD include a sudden drop in food intake, lethargy, and loosened cuticles with white spots 0.5 to 2.0 mm wide, visible beneath the carapace. In some cases, infected shrimp may exhibit a pink to reddish-brown color due to increased chromatophores. Additionally, white spots are occasionally observed in infected *P. vannamei* from America. WSSV can infect mesodermal and ectodermal cells, such as the subcuticular epithelium, in various crustacean species, leading to inconsistent mortality rates. The virus spreads through vertical and horizontal transmission, including cannibalism of infected dead shrimp and water-borne pathways. Furthermore, WSSV can reach uncontaminated areas via organisms exposed to contaminated effluents from shrimp farms. Vectors or reservoirs of WSSV include aquatic insect larvae, invertebrates, and copepods (Ganjoor, 2015).

##### 2.1.1.3 Yellow head disease (YHD)

Yellow head virus (YHV) genotype 1 is the causative agent of YHD. YHV is a rod-shaped, enveloped virus with +ssRNA, belonging to the family *Roniviridae* (Ganjoor, 2015). When tissues infected with YHV were observed under transmission electron microscopy (TEM), vesicles encapsulating virions were observed in the cytoplasm, and in the intracellular spaces. These virions were reported to have a diameter of approximately 40-50 nm and 150-200 nm in length (Walker and Mohan, 2009; Lightner, 2011). First cases of YHV were reported in Thailand in 1990 in a *P. monodon* culture, and eventually was spread widely in cultured shrimps all over the country (Cowley et al., 2000). Subsequently, YHD has been reported in cultured shrimps in Malaysia, Sri Lanka, India, Taiwan, Indonesia, Vietnam, and Mexico (Mohan et al., 1998; Wang and Chang, 2000; Walker and Mohan, 2009). Apart from *P. monodon*, other susceptible species to YHD include *L. vannamei*, *L. stylirostris*, *P. styliferus*, *Macrobrachium sintangense* and *Macrobrachium lanchesteri* (Ganjoor, 2015).

There are now eight recognized genotypes of YHV (Arulmoorthy et al., 2020). Gill associated virus (GAV) is the genotype 2, recognized as the Australian strain of YHV, belonging to the genus *Okavirus* in *Roniviridae* (Spann et al., 1997). GAV is a rod-shaped, enveloped, positive-strand RNA nidovirus (Cowley et al., 1999; Cowley and Walker, 2002). Infections caused by genotypes 3 to 6 were reported in *P. monodon* in Asia, Australia, and East Africa with no associated disease symptoms (Lee D et al, 2022). Furthermore, genotypes 7 and 8 were reported recently in diseased *P. monodon* (Mohr et al., 2015) and *F. chinensis* (Liu et al., 2014).

YHD in Asian intensive cultivation systems are considered as a dangerous *P. monodon* disease. However, YHV has also been reported to infect other species such as *P. aztecus*, *P. duorarum*, *P. japonicus*, *L. vannamei*, *P. setiferus*, and *P. stylirostris* (Stentiford et al., 2009). Although there is a higher chance for YHD-associated mass mortality in early to juvenile stages of shrimps, there is a chance that individuals in late post larval stages may also die due to infection (Limsuwan, 1991).

As the name “Yellowhead” implies, one of the major characteristics of this disease is yellowish or bleached appearance of the cephalothorax. Other gross signs include high feeding rates followed by a cessation in feed intake, and the presence of moribund shrimp along the pond’s edge (Stentiford et al., 2009; Walker and Mohan, 2009). Moreover, a reddish discoloration is observed in infected shrimps. Although GAV infection is identified as less severe due to low mortality, YHV can infect and cause necrosis in ectodermal and mesodermal tissue, especially in lymphoid organ and gills (Walker and Mohan, 2009). YHV is spread via horizontal transmission by cannibalism of moribund and weak shrimp, and vertical transmission by survivors of the disease, which suffer from persistent subclinical infections (Stentiford et al., 2009; Lightner et al., 2012).

##### 2.1.1.4 Infectious hypodermal and hematopoietic necrosis (IHHN)

IHHN is caused by IHHNV, which belongs to the family *Parvoviridae*. This virus is 22 nm in diameter, making it the smallest known virus to infect penaeid shrimps. IHHNV is a nonenveloped icosahedral virus with single-stranded DNA of 3.9 kb (Mari et al., 1993; Lightner et al., 2012; Ganjoor, 2015). IHHNV was first discovered in *L. vannamei* and *P. stylirostris* in America in the early 1980s, which then rapidly spread to Central America, Brazil, Mexico, Peru, Philippines, Thailand, Indonesia, Singapore, Malaysia (Lightner, 1996a) Australia (Krabsetsve et al., 2004), China (Arulmoorthy et al., 2020) and India (Rai et al., 2009).

Molecular testing identified significant variation within the IHHNV isolates from Asia (Tang et al., 2003; Krabsetsve et al., 2004; Tang and Lightner, 2006). However, American isolates showed lower sequence variation (99.6 to 100% identity) (Tang and Lightner, 2002) and 99.8% sequence identity to IHHNV isolates from the Philippines (Tang et al., 2003). Based on molecular and epidemiology studies of this virus, three different genotypes were identified: (I) Southeast Asia; (II) America/Philippines; and (III) East Africa, Madagascar, Mauritius, and Australia (Lightner, 1996a; Tang and Lightner, 2002; Tang et al., 2003; Lightner, 2011). The host species *P. monodon* and *L. vannamei* were only susceptible to IHHNV-I and IHHNV-II. Studies show that genetically diverse cultures of *P. monodon* belonging to the Indo-Pacific region carried an IHHNV-III DNA fragment in their genome, which prevented infection by the genotype III (Duda and Palumbi, 1999; Tang and Lightner, 2006).

In *P. stylirostris*, IHHNV can cause high mortality and low virulence in juvenile shrimps and adults, respectively (Motte et al., 2003). Gross signs of this viral infection in *P. stylirostris* include reduced food intake, changes in behavior, stunted growth, and appearance of white or buff-colored spots (these spots appear different from the white spots observed in WSSV-infected shrimps). These spots are often observed in the cuticular epidermis of the shrimp, making it appear mottled. In moribund shrimps of *P. stylirostris* and *P. monodon* suffering from the terminal stage of the infection, the mottled appearance changes to a bluish color with opaqueness in the abdomen (Brock and Lightner, 1990; Lightner, 1996a). In *L. vannamei*, “Runt-deformity syndrome” (RDS) is observed, which can be characterized by reduced growth and deformed cuticles (Duda and Palumbi, 1999). RDS in juvenile shrimps can also be distinguished by a bent or malformed rostrum, wrinkly antennal flagella, roughness and the appearance of ‘bubble-heads’ on the cuticles (Kalagayan et al., 1991; Browdy et al., 1993; Carr et al., 1996; Lightner, 1996a; Motte et al., 2003).

IHHNV infects the ectodermal and mesodermal tissues such as gills, hypodermis, connective tissues, nerve cord, lymphoid organs, and antennal gland (Lightner, 2011). This virus is spread via horizontal transmission through cannibalism and contaminated water (Lightner, 1996a), and vertical transmission through infected eggs (Motte et al., 2003).

##### 2.1.1.5 Infectious myonecrosis (IMN)

IMN is a novel viral infection caused by the Infectious myonecrosis virus (IMNV), which belongs to the *Totiviridae* family. IMNV is a non-enveloped icosahedral virus of 40 nm in size. The genome of this virus consists of a single double-stranded RNA (dsRNA) of 7,560 bp (Ganjoor, 2015). Two ORFs are present in the IMNV genome. ORF1 codes for capsid proteins and RNA-binding proteins, while putative RNA-dependent RNA polymerase is encoded by the second ORF (Poulos et al., 2006).

IMNV was initially discovered in *L. vannamei* of northeast Brazil in the year 2002, and later spread to countries in southeast Asia, including India and Indonesia (Senapin et al., 2007; Sahul Hameed et al., 2017). Genome sequencing analysis showed a 99.6% nucleotide sequence identity between the IMNV genomes from Brazil and Indonesia. This suggests that IMNV may have been transferred to Indonesia from Brazil in 2006 (Arulmoorthy et al., 2020).

IMNV can lead to cumulative mortality ranging from 40% to 70%. Experiments have demonstrated that *P. stylirostris*, *Fenneropenaeus subtilis*, and *P. monodon* can also be infected with IMNV, with shrimps at both juvenile and subadult stages being more susceptible (Arulmoorthy et al., 2020). Apart from increased mortality, lethargy, loss of coordination, and reduced food intake are gross signs of IMN. Furthermore, infected shrimps may appear at the water surface throughout the day and may exhibit necrotic regions in striated muscles that appears red (Nur’aini, 2009). In the acute phase of IMN, coagulative muscle necrosis can be observed with oedema of infected muscles, leading to fluid retention between muscle fibers and infiltration of hemocytes. The infection may further lead to hypertrophy due to lymphoid organ spheroids (LOSs) that generally appear in heart, gills, ventral nerve cord, and areas proximal to antennal gland tubules. LOS lesions are extremely consistent with IMNV lesions that are associated with acute, or chronic stages of the infection (Arulmoorthy et al., 2020). Primary target areas of IMNV include, striated muscles, hemocytes, lymph organ parenchyma cells, and connective tissues (Tang et al., 2005). IMNV is spread via horizontal transmission through cannibalism of the infected shrimps and contaminated water (Poulos et al., 2006; Lightner, 2011).

##### 2.1.1.6 Monodon baculovirus disease (MBD)

MBD is caused by a rod-shaped, enveloped virus which is known as Monodon baculovirus (MBV). This virus belongs to the *Baculoviridae* family and has circular dsDNA, with a genome size between 80 and 160 Kbp (Mari et al., 1993; Ganjoor, 2015). The first cases of MBD were discovered in 1977 in *P. monodon* shrimp farms in Taiwan. Subsequently it spread to other countries such as Australia, Philippines, China, Malaysia, Indonesia, Sri Lanka, South Africa, and USA. MBV is also known as *P. monodon* singly enveloped nuclear polyhedrosis virus (PmSNPV). Apart from MBV, two SNPV strains have been reported. They are the plebejus baculovirus and benettae baculovirus (Arulmoorthy et al., 2020).

Although the primary host species of MBV is *P. monodon*, multiple cases have been reported from other species including *Macrobrachium rosenbergii*, *Penaus penicillatus*, *Penaeus semisulcatus*, *Metapenaeus ensus*, *P. merguiensis*, *Penaeus esculentus*, *L. vannamei*, and *Penaeus kerathurus* (Lightner et al., 1987; Doubrovsky et al., 1988; Lightner, 1988; Chen et al., 1989; Colorni, 1989; Vijayan et al., 1995; Lightner, 1996a, 1996; Rajendran et al., 2012). The most susceptible individuals for MBV are larval and juvenile shrimps. However, the disease has been observed in all developmental stages of *P. monodon* (Brock and Lightner, 1990). This viral infection causes anorexia, subsequent retarded growth (Ganjoor, 2015), lethargy, and fouling of the surface with darkened appearance. Moreover, when compared to healthy individuals, the infected shrimps are smaller. MBV targets the anterior midgut and the hepatopancreas (Lightner, 1993). The virus spreads through horizontal transmission via the fecal oral route (Chen et al., 1992).

#### 2.1.2 Bacterial diseases

##### 2.1.2.1 Necrotizing hepatopancreatitis (NHP)

NHP is a bacterial disease caused by *Hepatobacter penai*, a bacterium like *Rickettsia*. This is a Gram-negative, dimorphic bacterium found in the cytoplasm of infected hepatopancreatic cells. The rod-shaped rickettsia-like body (0.35-0.9 μm) is the most common form. The spiral (helical) form (0.25 x 2-3.5 μm) possesses eight flagella at the basal apex (Lightner et al., 1992). The first cases of NHP were discovered in *L. vannamei* cultures in Texas, in the year 1985. Later in 1993, a similar disease surfaced in Peru, which was later confirmed as NHP through molecular diagnostic methods such as polymerase chain reaction (PCR) and restriction fragment length polymorphism (RFLP) (Loy et al., 1996). The isolates were further analyzed to have morphologies that are extremely similar, to be considered as identical. With a cumulative prevalence of 39.3%, Latin American countries have reported the highest number of NHP cases (Cuéllar-Anjel et al., 2018). NHP caused mass mortality in countries such as Peru, Costa Rica, Venezuela, Mexico, Panama, and Brazil. In the year 2006, Mexico also encountered an NHP outbreak (del Río-Rodríguez et al., 2006). NHP disease is more likely to occur in regions with high water temperature (29-35°C) and salinity (30-40 ppt) (Lightner, 1996a).

Primary host species susceptible to NHP are *F. aztecus*, *L. vannamei*, *F. californiensis*, *P. setiferus*, and *P. stylirostris*. The gross signs of NHP consists of reduced food consumption, slow growth, anorexia, softened shells, flaccid bodies, and expanded chromatophores, along with fouling of the body surface (Lightner, 1996a). NHP generally occurs in hepatopancreatic cell types (Lightner et al., 2012) and can be spread via horizontal transmission through cannibalism of infected tissue (Vincent et al., 2004).

##### 2.1.2.2 Vibriosis

Vibriosis is one of the major diseases that affect shrimp aquaculture farms and has been associated with mortality of shrimp cultures around the globe. Vibriosis is caused by Gram-negative bacteria, and multiple species may be associated with the disease. In this regard, species that have caused vibriosis include *Vibrio harveyi*, *Vibrio alginolyticus*, *Vibrio splendidus*, and *Vibrio parahaemolyticus*. These bacteria belonging to the *Vibrionaceae* family, have caused mortality in *P. monodon* larvae in shrimp farms. It has been demonstrated that only some isolates of *V. harveyi* have shown to possess virulence, indicating molecular and genetic variation. The over-intensification of shrimp aquaculture may have been associated with the emergence of vibriosis, as the disease has been known to occur in shrimps in stressful conditions (Ishimaru et al., 1995; Lavilla-Pitogo and de la Pena, 1998).

*V. harveyi* is a luminous bacterium, hence it is visible in infected shrimps at nighttime. Gross signs of this disease include reduced growth rate, lethargy, opaque muscles, and presence of patches (Karunasagar et al., 1994). Furthermore, necrosis of the appendages, gut emptiness, and expansion of chromatophores can also be observed in vibriosis-infected larvae (Chandrakala and Priya, 2017).

##### 2.1.2.3 Acute hepatopancreatic necrosis disease (AHPND)

AHPND is a bacterial disease which is recognized as a major threat to shrimp aquaculture (Tran et al., 2013). In 2009, China reported an outbreak in shrimp cultures which was diagnosed as AHPND (Nunan et al., 2014). Subsequently, Malaysia, Philippines, Mexico, Vietnam, Thailand (Shinn et al., 2018), Bangladesh (Eshik et al., 2017), and the United States (Dhar et al., 2019) also encountered AHPND outbreaks which caused huge losses to their shrimp aquaculture productivity.

AHPND is caused by strains of *V. parahaemolyticus*, which are highly omnipresent, opportunistic, marine bacteria (Tran et al., 2013). The Gram-negative bacteria belonging to the family *Vibrionaceae*, contains the plasmid pVa1, which causes virulence. This plasmid carries transposons, binary toxin genes and conjugate transfer genes. This indicates that it is possible for plasmid transfer to other strains of this bacteria or other species (Lee et al., 2015).

This bacterium infects the shrimp by colonizing the gut (Tran et al., 2013; Lai et al., 2015). The pVa1 plasmid then expresses the binary toxins that invade the hepatopancreas (Prachumwat et al., 2019) and trigger shedding of the epithelial cell lining of the tubule (Tran et al., 2013). This makes the hepatopancreas appear pale. The severity of the disease can be influenced by the gut microbiota of the shrimp. When infected, the bacterial communities residing in the gut and hepatopancreas are exposed to an imbalance in their local distribution, known as dysbiosis (Chen et al., 2017). On the other hand, the host can gain protection against the pathogen by the enrichment of certain bacterial species (Yu et al., 2018).

Penaeid shrimp species that have been identified as highly susceptible hosts to AHPND-causing bacteria include *L. vannamei* and *P. monodon* (Tran et al., 2013; Zorriehzahra and Banaederakhshan, 2015). Gross signs of infected shrimps include exhibition of shrunken and pale hepatopancreas, gut emptying, low feed intake, swimming spirally, and sluggishness (Zorriehzahra and Banaederakhshan, 2015). This disease is spread via horizontal transmission through co-habituation or ingestion of the pathogen. In this regard, cannibalism of infected and dead shrimp, fecal-oral transmission, and feed pellets colonized by the bacteria can also spread AHPND (Tang et al., 2020).

#### 2.1.3 Fungal diseases

Like viruses and bacteria, fungal pathogens such as *Lagenidium callinectes* and *Sirolpidium* spp., have been known to cause diseases in penaeid shrimps as well. Generally, fungal infections are found in larval stages of the shrimps with gross signs including lethargy, presence of mycelia and fungal spores, especially in appendages and gills. Larval mycosis and Fusariosis are common fungal diseases of penaeid shrimp (Karunasagar et al., 2004). A recent study on a new biofloc system also reported that a *Fusarium verticilliodes* infection resulted in cumulative mortalities in white leg shrimps (Hussein et al., 2024).

##### 2.1.3.1 Larval mycosis

Larval mycosis is a fungal disease caused by *Haliphthoros philippinensis*, *Lagenidium callinectes*, *Sirolpidium* sp., and *Lagenidium* sp. This disease can affect *P. monodon* eggs, larvae, and post-larvae. In shrimps infected by *Lagenidium*, vesicles with zoospores of high motility are formed at the end of the discharge tube, invading the host. On the other hand, when affected by *Haliphthoros*, vesicles are not formed, but long discharge tubes are observed. In contrast, *Sirolpidium* infection results in short discharge tubes, and vesicles do not form either (Baticados et al., 1990).

Significant signs of infection include whitish appearance, weakness and high risk of death. Moreover, the mortality rate may reach 100% within 2 days. The inner tissue of the individual is replaced by the zoospores, with discharge tubes protruding out from the body. If the egg is infected, they are prevented from hatching and respiratory difficulties are observed in infected larvae (Baticados et al., 1990).

##### 2.1.3.2 Fusariosis

Fusariosis is caused by *Fusarium* spp., such as *Fusarium solani*, which are opportunistic soil fungi that have been reported to infect penaeid shrimps. Cases of fusariosis has been found in *P. californiensis*, *L. vannamei* and *P. stylirostris* from Mexico (Lightner et al., 1979). Moreover, cultivated *P. japonicus* shrimps from Japan and France have also been affected by this disease (Bian and Egusa, 1981; Criado-Fornelio et al., 1988). Gross signs of this infection include large melanized lesions on cephalothorax and abdomen, with degradation and ulceration of cuticles (Colorni, 1989). Moreover, fungal hyphae can also be observed in infected tissue, under a light microscope (Karunasagar et al., 2004).

#### 2.1.4 Protozoan diseases

##### 2.1.4.1 Microsporidiosis

Microsporidiosis is caused by an endoparasite, Microsporidia. This protozoan has been shown to infect shrimps belonging to the species *P. indicus*, *P. monodon*, and *P. merguiensis*. Generally causing infection in juveniles and adult shrimps, the infected regions can be characterized by an opaque white appearance. The pathogen is known to form spores in infected tissue and may lead to white ovaries, which can cause sterility in spawners. Though the infection rate was estimated to be as low as < 10%, Microsporidia have been known to be extremely pathogenic (Baticados et al., 1990).

##### 2.1.4.2 Gregarine disease

Gregarine disease is caused by gregarine, which are usually present in the gut of penaeid shrimps. They can infect *P. monodon* shrimps at larval, post larval, juvenile, and adult stages. When gregarines are prevalent, they can interfere with functions of the hepatopancreatic duct, such as particle infiltration. Gregarine infection rates up to 94% have been reported in shrimp cultures (Baticados et al., 1990).

### 2.2 Microbiome of penaeid shrimps

‘Microbiota’ refers to the bacterial community that resides and maintains a symbolic or commensal relationship with a host organism. The genetic material or metagenome linked with a certain microbiota is known as the ‘Microbiome’ (Kumar et al., 2020). They function as an endocrine organ by serving as a barrier against pathogenic invasion and stimulating the acquisition of host nutrients through several metabolic pathways (Xiong et al., 2017). In penaeid shrimps, the composition of gut microbiota can be influenced by both intrinsic and extrinsic factors. This includes, the diet of the shrimp, consumption of probiotics, physiochemical properties of the water and the growth stage (Xiong et al., 2015b; Yan et al., 2016; Vargas-Albores et al., 2017; Garibay-Valdez et al., 2019).

In shrimp aquaculture systems, shrimp share the ecosystem with invading pathogens. Some studies indicate that the intricate interaction between the shrimp, environmental factors, and the microbiota of the surroundings may cause certain pathogenic diseases to emerge (Boutin et al., 2013; Xiong et al., 2015a). Moreover, the severity of the disease in shrimps is correlated with the composition of gut microbiota (Xiong et al., 2015b). On the other hand, recovery of the gut bacterial composition has shown to increase survival rate in shrimps (Rungrassamee et al., 2016). Hence, it can be concluded that the bacterial community in the intestine is a good indicator of the status of shrimp health (Round and Mazmanian, 2009; Clemente et al., 2012).

**FIGURE 1:**
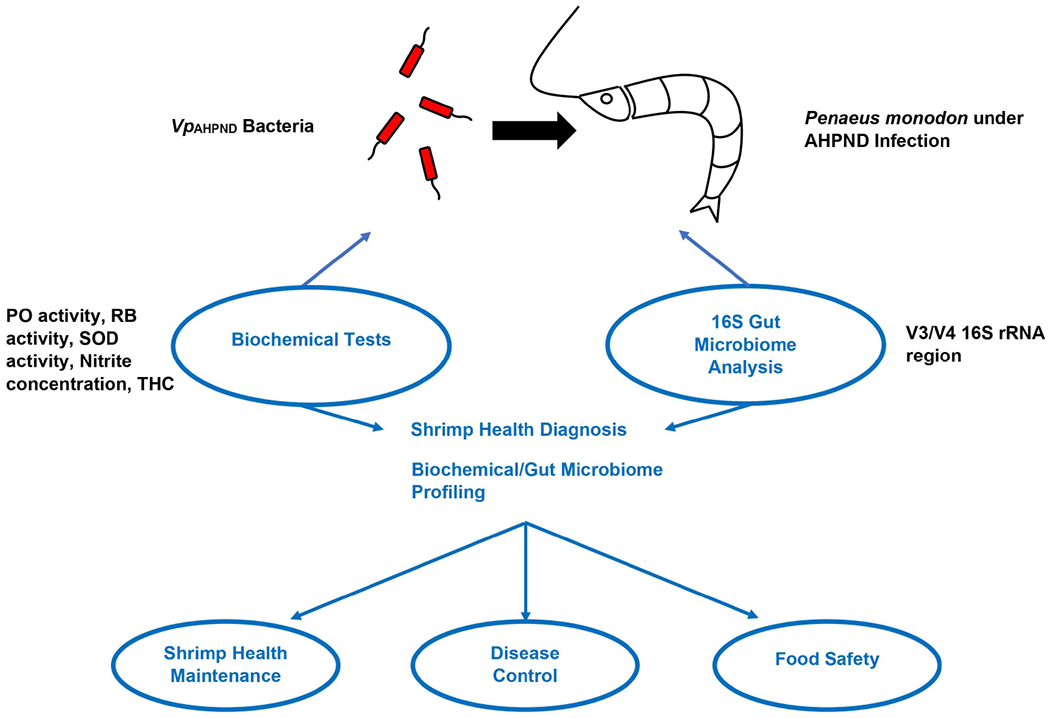
Diagnostic methods for Penaeus monodon health during AHPND infection. *Note: Adapted from “Biochemical indexes and gut microbiota testing as diagnostic methods for Penaeus monodon health and physiological changes during AHPND infection with food safety concerns”* (Soo and Bhassu, 2022).

The microbiome of penaeid shrimps is dominated by Gram-negative bacteria belonging to the phylum Proteobacteria (Holt et al., 2021). In *P. monodon* and *L. vannamei*, the gut microbiota mostly comprises of *Vibrio* and *Photobacterium* spp., which belong to the class Gamma-proteobacteria (Chaiyapechara et al., 2012; Rungrassamee et al., 2013; Tzuc et al., 2014; Rungrassamee et al., 2016; Zheng et al., 2016). Apart from this, other phylum including Actinobacteria, Bacteroidetes, Firmicutes and Fusobacteria are also a part of shrimp microbiota (Rungrassamee et al., 2013). With recent technological advancements, using the gut microbiome as a tool to address gut microbiota in various shrimp populations from farm to table will ensure that food safety and hygiene are achieved. This is in line with sustainable development goal strategies, which ensure the fast detection of gut microbiome diversity. Later, strategies using control measures to reverse the pathogens will enable quick control. The intestinal microbiota is crucial for the diverse host physiological processes, such as immunity growth, metabolism upkeep (Lin and Zhang, 2017; Thursby and Juge, 2017), pathogen defense, health maintenance, and nutrient absorption (Blancheton et al., 2013). Following the widespread application of high throughput next-generation sequencing (NGS) technology, there has been an increase in NGS application in 16S rRNA-based microbial community analysis due to cost and effectiveness concerns (Caporaso et al., 2011). These studies are vital for the identification of microbial diversity (Ritchie et al., 2008), trend of microbial gene content (Konstantinidis and Tiedje, 2005), and their correlation to host or environmental parameters (Hamady and Knight, 2009). A study conducted by Soo and Bhassu, 2022 showed that both biochemical tests and 16S rRNA analysis can be proposed as a combined strategy for shrimp health diagnosis, ensuring shrimp health maintenance, disease control, and food safety. This was illustrated in a graphical abstract (Figure 1) that shows the simplified method on how gut microbiome can serve as biomarkers for healthy shrimps.

16S rRNA V3/V4 hypervariable region is commonly selected in 16S amplicon sequencing analysis as shown previously (Kommedal et al., 2008; Edwards et al., 2012; Derakhshani et al., 2016; Wu et al., 2016). 16S amplicon sequencing analysis is also advantageous due to its lesser reliance on the quality of extracted DNA samples (Rintala et al., 2017). In spite of the widespread application of 16S amplicon sequencing technology in recent years, there have only been relatively few publications reported for the shrimp aquaculture industry, especially those involving diseased conditions (Rungrassamee et al., 2016; Zheng et al., 2016; Pilotto et al., 2018).

The composition of penaeid shrimp microbiota is different at various developmental stages. For instance, *Photobacterium* spp. is found abundantly (80%) in *P. monodon* shrimps at post-larval stages, while *Vibrio* dominates the guts of juvenile shrimp. Other bacteria such as *Fusobacteria* are observed in PL15, while Spirochaetes are only present in J2 shrimps. Furthermore, while *Actinobacteria*, are observed in both post-larval and juvenile stages, *Listonella* is only found in juvenile stages (Rungrassamee et al., 2013). In *L. vannamei*, similar trends were observed, where *Photobacterium* spp. and *Vibrio* spp. dominated in the early stages of life, while *Actinobacteria* were more abundant in the adult stages (Cicala et al., 2020). The differences in bacterial diversity at various life stages may also be associated with differences in diet composition at different life stages. At the post-larval stage, the shrimps are given live feed, while at the juvenile stage, they are fed commercial pellets (Rungrassamee et al., 2013).

The changes in the microbiome may influence the onset and development of pathogenic diseases (Holt et al., 2021). To fight against bacterial diseases, antibiotics and probiotics have been used in shrimps to modulate the microbiota and develop resistance against specific pathogens. However, the use of antibiotics in shrimps, is restricted to avoid the development of antibiotic-resistance in prevailing bacterial pathogens. Hence, developmental strategies are required to reduce the occurrence of disease outbreaks, decrease the usage of antibiotics, and reinvigorate the health of the shrimp. Consequently, it is crucial to understand the association between the microbiota and shrimp immune system. Additionally, enhancing on the microbial dynamics of the shrimp microbiome and the ecosystem during disease outbreaks is also important (Kumar et al., 2020). For instance, if the gut microbiota is maintained, the risk of diseases caused by opportunistic pathogens in shrimps could be reduced (Rungrassamee et al., 2013).

### 2.3 Management and diagnostic approaches of penaeid shrimp diseases

Penaeid shrimps do not have an adaptive immune system that can facilitate natural protection against various pathogens and allow for immunization against viruses via standard vaccination (Arala-Chaves and Sequeira, 2000; Johnson et al., 2008). The main objectives of the shrimp health administration in aquaculture or disease management techniques are thus to exclude pathogens, and to avoid stressful environmental conditionals that might favor the emergence and spread of diseases (Walker and Mohan, 2009). This includes implementation of a structured biosecurity at shrimp farms, breeding programs for SPR^1^ or Specific pathogen free (SPF) stocks, the use of probiotics, and the development of pathogen detection and diagnostic methods. Hence, bacterial species, such as *Lactobacillus* or *Nitrobacter* help to improve survival rate, water quality, immunity, and disease resistance through space competition with disease-causing bacteria, such as *Vibrio* spp., as demonstrated in a recent study conducted by Amiin et al. (2023). The use of prebiotics, probiotics and synbiotics are key ingredients to maintain shrimp gut health at optimum levels throughout the production cycle, ensuring high survival and growth (Noman et al., 2024).

#### 2.3.1 Biosecurity in shrimp aquaculture

Biosecurity practices involves strategies aimed at excluding certain pathogens from shrimp farms to prevent the occurrence of diseases (Walker and Mohan, 2009). It is imperative to establish effective biosecurity measures during all stages of shrimp cultivation, from breeding to harvest, to prevent pathogen entry or release into the environment. There are multiple measures that are implemented on-farm to ensure maximum biosecurity. Firstly, the aquaculture habitat or ponds are initially prepared by drying and exposing them to the sun to eradicate residual pathogens. Secondly, the water is filtered and disinfected before the shrimps are stocked or exchanged during grow-out stage (Walker and Mohan, 2009). The water quality is also frequently monitored and controlled by carrying out tests such as Water Quality Index (WQI), which can allow for the qualitative estimation of shrimp diseases in case of an outbreak (Xiong et al., 2016). Moreover, fences and nets are built to prevent potential carrier organisms and birds from feeding on or spreading infected or moribund shrimps. Furthermore, the effluent from the farm is treated before discharge to avoid the releasing of contaminated water into the ecosystem (Fegan and Clifford, 2001; Vanpatten et al., 2004; Subasinghe, 2005). In addition, shrimps are monitored during all stages of life cycle for early signs of infections by carrying out frequent histological examinations (Walker and Mohan, 2009).

In medium to large semi-intensive shrimp farms, the management of diseases are carried out at a higher level compared to small-holder farms. In more equipped and semi-intensive farms, aspects such as the aquaculture pond and land, treatment of wastes, water usage and maintenance records are managed more effectively. Moreover, they restrict the import of broodstocks from the wild and reduce usage of chemicals and antibiotics in the shrimp farms (Walker and Mohan, 2009). On the other hand, low-income farms have limited awareness, education, and resources to comprehensively practice disease management strategies (Padiyar, 2009). Hence, Better Management Practices (BMP) were developed. BMP are more affordable and effective measures that can be implemented by small-holder farmers to reduce shrimp diseases. The main objectives of BMP are to reduce the risks associated with health problems in shrimps, thereby maximizing the efficiency and production. Moreover, it also aims to minimize the adverse effects of shrimp aquaculture on the environment and enhance safety and quality of cultivated shrimps. Furthermore, BMP are also implemented by small producers to improve social benefits, acceptance, and sustainability of shrimp cultivation (Walker and Mohan, 2009).

The use of artifical intelligence (AI) and machine learning (ML) aided by molecular images are the latest technologies to understand the disease outbreaks in recent decades, necessitating an AI and ML approach. A study conducted by Ping and Liem (2000), attempted to predict shrimp disease occurrence using artificial neural networks versus logistic regression. However the study was inconclusive due to differences in farm and pond management practices. The integration of advanced technologies such as image-based machine learning, augmented reality (AR), surface-enhanced Raman scattering (SERS), and sensor technology, coupled with Internet of Things (IoT), big data, AI, 5G networks, cloud computing, and robotics is expected to have a high impact on disease management in aquaculture (Malik et al., 2017a; Islam et al., 2024; Malik et al. 2017b; Jothiswaran et al., 2020; Li and Li, 2020).

#### 2.3.2 Specific pathogen free stocks

To reduce the impact of diseases on shrimp aquaculture productivity and to guarantee sustainability, it is crucial to acquire high-quality seed or post larvae to stock shrimp farms (Walker and Mohan, 2009). The deployment of SPF stocks has been an emerging trend in the shrimp aquaculture sector and has been recognized as an effective strategy to control shrimp stocks to enhance biosecurity in shrimp farms (Alday Sanz et al., 2020; Walker and Mohan, 2009; Turkmen and Toksen, 2010). SPF stocks are cultured under strict quarantine and screening at breeding centers to acquire shrimp populations devoid of specific or more pathogens. These pathogens should meet a certain criterion. In this regard, the pathogen should be effectively diagnosed and physically removable from the farm. In addition, the pathogen should be classified as a significant threat to the shrimp aquaculture industry (Lotz, 1997; Lightner, 2003).

Currently, SPF stocks that are free of WSSV, YHV, IHHNV, TSV and IMNV are available. However, on a large scale, SPF populations of only *L. vannamei* are currently obtainable (Walker and Mohan, 2009). It is significant to note that SPF stocks are not resistant to diseases or free of diseases. This is because there is a high chance that they might be infected with a known pathogen that is not listed by the breeding center. On the other hand, the stocks may also be infected by an unknown pathogen. Additionally SPF lists does not include the genus *Vibrio*, even though they can potentially cause shrimp diseases. *Vibrio* resides in the gut microbiome of shrimps, and hence cannot be physically eliminated from shrimp farms. SPF shrimps lack innate resistance to pathogens; therefore, disease resistant shrimps can only be bred into a line via selective breeding (Walker and Mohan, 2009).

#### 2.3.3 Specific pathogen resistant stocks

SPR^1^ stocks are refractory to pathogenic infections and when exposed to such pathogens, they do not exhibit any gross symptoms of the disease. The difference between SPF and SPR^1^ stocks is that SPF relates to an individual’s health, whereas SPR^1^ pertains to its genetic status (Alday-Sanz et al., 2020). Followed by the development of SPF stocks, in 1990, shrimp breeding programs were established to selectively breed shrimp stocks with commercially desirable traits such as resistance to certain diseases. These breeding programs were based on the understanding of the quantitative genetics of penaeid shrimps (Walker and Mohan, 2009).

The development of SPR^1^ stocks were more focused on certain viral shrimp diseases that inflicted major economic losses to the sector. They include TS, IHHN, and WSD (Argue et al., 2002; Kong et al., 2003; Jiang et al., 2004; Gitterle et al., 2005; Cuéllar-Anjel et al., 2011). In the past two decades, many researchers have managed to enhance penaeid shrimp resistance to TSV via selective breeding (White et al., 2002; Kong et al., 2003). In this regard, *L. vannamei* stocks that have been selectively bred over 15 generations, were reported to exhibit 100% survival followed by TSV infection (Moss et al., 2011). On the other hand, for WSSV, minor improvements have been made in developing SPR^1^ stocks. In 2005, a mean selection of 2.8% (Gitterle et al., 2005) was reported in *L. vannamei*, while a 22.7% survival rate was reported in 2010 (Huang et al., 2011). In addition, in 2011, survival rates between 23% to 57% against WSSV were reported in *L. vannamei* (Cuéllar-Anjel et al., 2011).

#### 2.3.4 Probiotics management for shrimp growth

The use of antibiotics against bacterial pathogens in shrimp farms have led to the development of antibiotic-resistant bacteria, decreasing the efficiency of antibiotics (Vaseeharan and Ramasamy, 2003). Moreover, other traditional approaches such as the use of disinfectants to eliminate all microbiota in the aquaculture pond (Summerfelt et al., 2009) have led to imbalance in microbial community which decreases the competition within the niche and provides the opportunity for opportunistic pathogens to multiply. Examples of such bacteria include *Vibrio* species (Attramadal et al., 2012). Hence, it has been advised to avoid the use of antibiotics and disinfectants in shrimp aquaculture systems. Consequently, the application of probiotics was suggested, and some farmers have been adding probiotics to shrimp cultivation ponds as a control strategy against bacterial pathogens (Flegel et al., 2008).

Probiotics are live microorganisms that are orally administered as feed supplements, to enhance the balance of gut microbiota (Newaj-Fyzul et al., 2014). Some studies have suggested that the composition of gut microbiome is different in diseased shrimps, compared to their healthier counterparts (Xiong et al., 2015a; Zheng et al., 2016). These studies indicated that most common species found in healthy shrimp gut are known and have been used as probiotics, whereas the species of microbes isolated from diseased shrimps are labeled as potential pathogens (Zheng et al., 2016). The use of probiotics in shrimp health management has been reported to enhance the immune response and decrease the prevalence of *Vibrio* species (Li et al., 2007).

Multiple studies have validated the successful application of probiotics in shrimp disease management (Li et al., 2007; Cao et al., 2015; Solanki et al., 2015). Generally, the application of probiotics is standardized throughout the shrimp life cycle, although studies have shown that susceptibility to potential pathogens may vary based on the developmental stages (Zheng et al., 2016). This may limit the extent of probiotic effectiveness (Wang et al., 2008; Ninawe and Selvin, 2009). A study counter-indicated that probiotics do not improve the survival rate and productivity of shrimp aquaculture. Moreover, it is recommended to tailor the probiotic administration to the composition of intestinal microbiota at different developmental stages of the shrimp life cycle (Xiong et al., 2015a).

#### 2.3.5 Currently available diagnostic methods

For effective management of penaeid shrimp diseases, the availability of rapid, sensitive, convenient, and reliable diagnostic methods is significant for early diagnosis and prevention of the spread of disease. The most commonly available diagnostic methods include histological methods such as hematoxylin and eosin (H&E) staining, light microscopy, transmission electron microscopy (TEM), and fluorescent microscopy. Modern diagnostic and research laboratories for penaeid shrimp rely on traditional methods adapted from fish, veterinary, and human diagnostics. These include case history analysis, gross signs and behavioral observation, morphological pathology using bright-field or phase-contrast light microscopy, electron microscopy, and classical microbiology techniques (bacteriology and mycology). However, techniques involving tissue and cell culture, hematology, and clinical chemistry, which are staples in vertebrate diagnostics, have either been unsuccessful or provided unreliable diagnostic data for shrimp (Lightner and Redman, 1998). In contrast, serological methods using polyclonal and monoclonal antibodies, and molecular methods like gene probes and Polymerase Chain Reaction (PCR), have proven to be accurate and standardizable for disease diagnosis and pathogen detection in penaeid shrimp, particularly for certain viruses. It is crucial to understand the workflow from classical methods to the latest diagnostic techniques, including new AI methods in molecular imaging, understanding cellular events within the shrimp innate immune system, and applying the latest technologies such as single-cell sequencing.

In addition, molecular methods such as PCR, nested PCR, multiplex PCR, multiplex reverse transcription-PCR (mRT-PCR), real-time RT-PCR, multiplex RT-nested PCR, and *in-situ* DNA hybridization methods are also very common. Furthermore, monoclonal antibody based tests such as ELISA, dot-blot assay, and lateral flow chromatographic assay are also shown to be efficient diagnostic methods of shrimp pathogens (Ganjoor, 2015). In this regard, lateral flow chromatographic assay strips have been developed for the detection of certain pathogens such as WSSV and are being applied in Japan and Thailand (Flegel, 2006). These strips were also used by unskilled farmers to diagnose shrimp pathogens immediately at the pond site, without the need for technical expertise (Flegel et al., 2008). Additionally, a similar rapid lateral flow immunoassay test kit for WSSV detection has also been developed commercially in Taiwan (Hsu et al., 2022). On the other hand, methods such as PCR and RT-PCR require sophisticated equipment and skilled personnel to operate them (Flegel et al., 2008; Ganjoor, 2015). Moreover, loop-mediated isothermal amplification (LAMP) in combination with amplicon detection via chromatographic lateral flow dipsticks (LFD) has also been recognized as an easier detection method (Nimitphak et al., 2008). Recently, nanotechnology has been applied in shrimp pathogen diagnosis through nanopores, protein arrays, nanoarrays, nanoparticles, nano-vaccines, and nano-based sensors (Govindaraju et al., 2020).

#### 2.3.6 Contribution of next-generation sequencing in genomic studies

NGS technologies were developed in the early 2000s, transforming the field of biological sciences. These DNA and RNA sequencing technologies have revolutionized the studies of OMICS, allowing novel directions in relevant fields of research that have never been considered in the past (Moorthie et al., 2011). NGS is a high-resolution technology that is cost-effective and efficient. Millions of fragments of DNA can be sequenced concurrently in a short period of time. Examples of NGS platforms include Illumina or Solexa, Roche, Helicos, ABI SOLiD, and Oxford Nanopore (Slatko et al., 2018). These technologies have provided exceptional opportunities for high-throughput applications in the field of functional genomics research (Moorthie et al., 2011).

Conventional approaches for the detection of pathogens are time-consuming and laborious. Through NGS platforms, various information regarding pathogenic diseases can be interpreted. For example, genome sequencing allows for the identification of species, strain of pathogens, its virulence, and the mechanism of pathogenesis. Furthermore, studies on molecular epidemiology and antibiotic-resistance of a pathogen can also be carried out using this technology (Fournier et al., 2014). For instance, Oxford nanopore (Oxford, UK) developed a DNA/RNA sequencing device that can be plugged to a computer and connected to central databases for the assembly and analysis of sequences using the internet. This allows for the detection and analysis of pathogens in real-time at shrimp farms. Moreover, these approaches will also be able to detect unanticipated nucleotide sequences belonging to new or currently unidentified strains of pathogens (Flegel, 2019).

Metagenomic approaches can be used to profile microbial communities present in the aquaculture system (Martínez-Porchas and Vargas-Albores, 2017). The data obtained from these studies can be used to understand the interactions between existing microbes and invading pathogens, that are both synergic and antagonistic. These studies can be applied in developing more cost-effective control strategies for disease management in shrimps. Simultaneously, NGS technologies has also made studies on environmental DNA (eDNA) of shrimp aquaculture systems possible (Shaw et al., 2016). The interaction between organisms in the ecosystem, lead to shedding of eDNA. eDNA is a significant tool that can be applied in ecological studies to monitor the biodiversity and to detect invading species (Barnes et al., 2014; Goldberg et al., 2016). These studies can be used to identify the optimum microbial community in the aquaculture systems for enhanced productivity and long-term sustainability (Flegel, 2019).

The advancement of NGS platforms has also been applied in the establishment of new breeding programs. Marker-assisted selection (MAS) allows for the selection of individuals with economically desirable traits, based on genetic markers, and has shown to increase the efficiency of breeding programs (Shekhar et al., 2021). Selective breeding for enhanced growth has increased the productivity of *L. vannamei* in the shrimp aquaculture industry. Other determinants of cultivated shrimp productivity include traits that have low heritability and are difficult to measure, such as resistance to diseases (Yáñez et al., 2015). NGS offers platforms for low-cost whole genome (DNA) and transcriptome (RNA) sequencing that can be used to identify genetic markers for traits of interest (Bayliss et al., 2017).

To enhance the sustainability of penaeid shrimp culture, it is important to understand the genomic architecture of the complete genome of penaeid shrimps. However, due to the presence of large number of repeat sequences, it has been challenging to generate a reference genome for all shrimp species (Rodriguez-Anaya et al., 2018). Nevertheless, owing to NGS technologies, a remarkable progress has been made to the advancement of other molecular genetic resources, such as generation of genetic maps, transcriptomes, and identification of qualitative trait loci (QTL) (Shekhar et al., 2021). For instance, the genome sequence and draft assembly of *M. japonicus* and *P. monodon* genomes have been generated with sequencing data up to 132.86 Gb and 132 Gb, respectively (Yuan et al., 2018). Recently constructed linkage maps of shrimps allowed for the identification of QTL that are economically important. They include shrimp weight and length of the body in *L. vannamei* (Andriantahina et al., 2013; Yu et al., 2015), resistance to WSSV in *P. monodon* (Robinson et al., 2014) and sex linkage in *P. monodon* (Staelens et al., 2008; Guo et al., 2019). Moreover, QTL related to high pH tolerance has also been mapped, very recently (Huang et al., 2020).

RNA sequencing (RNA-Seq) via NGS platforms has allowed for the development and implementation of transcriptomic analysis of *L. vannamei*. The potential functional genes and subsequent proteins that are engaged in the route of *V. parahaemolyticus* infection have been identified from the transcriptome analysis of pathogen-free larvae of *L. vannamei*. These results indicated that the immune response of the shrimp has evolved against *Vibrio* infection (Li et al., 2012). Moreover, the identification of gene expression involved in immune response against other pathogens such as TSV (Sookruksawong et al., 2013), and WSSV (Chen et al., 2013; Peruzza et al., 2019, 2020) has also been made possible by using transcript profiling data.

Concurrently, RNA-Seq has been used to generate the transcriptome of gonads in *L. vannamei* reproductive systems. These data were used to identify genes that are expressed in metabolic routes of the reproductive system of shrimp including sexual differentiation, ovarian follicle growth, development and maturation of gonads and oocytes (Peng et al., 2015). These results can further be applied in MAS for the selection of high-quality stocks with inheritable traits that can improve growth, nutrition, maturation, tolerance to various environmental stress and tolerance against pathogens (Rodriguez-Anaya et al., 2018).

To sum up, NGS platforms have enhanced the application of OMIC technologies in the field of shrimp disease management research. Further advancement of these technologies may be a key to increase the sustainability of shrimp aquaculture industry.

## 3 Microbial control strategies

### 3.1 Improvement of pathogen detection methods

The development of rapid, reliable, and convenient diagnostic or detection methods is a significant aspect of penaeid shrimp disease control programs. The most reliable and commonly used diagnostic methods such as PCR-based methods are not readily available for diagnosis in shrimp aquaculture farms. Moreover, these methods require advanced equipment and skilled personnel to operate such machines and interpret data (Flegel et al., 2008). Hence, there is a high demand for point-of-care (POC) methods that farmers can use at pond side, such as the recently developed lateral flow chromatographic immunodiagnostic strips (Flegel, 2006).

There have been several advancements in the development of early disease detection technologies. In the past few years, researchers have developed a method called Sensitive High Efficiency Reporter unLOCKing (SHERLOCK). This method is highly sensitive, rapid and was able to develop lyophilized paper strips for the diagnostic testing of human Zika and Dengue virus. This POC method allowed the device to be used in places with no power or infrastructure (Gootenberg et al., 2017, 2018; Myhrvold et al., 2018). With the increase in knowledge and innovation in Clustered Regularly Interspaced Short Palindromic Repeats (CRISPR) technology, this tool has been applied in many fields of scientific research. In 2019, a CRISPR-based SHERLOCK diagnostic method was developed which allowed for accurate single copy detection of WSSV in penaeid shrimps. This assay is rapid and highly sensitive with potential applications in early detection of penaeid shrimp viruses at pond side (Sullivan et al., 2019). The recent study by Major et al. (2023) successfully adapted a SHERLOCK assay, initially designed for human diagnostics, to detect RNA and DNA pathogens in shrimp. This innovative RT-LAMP CRISPR/Cas diagnostic assay targets TSV and WSSV in *L. vannamei*, offering significant potential for field-deployable applications. This breakthrough is set to enhance biomonitoring in shrimp aquaculture and lays the groundwork for developing rapid and efficient diagnostics in the broader agricultural sector.

Concurrently, advanced multiplex PCR methods have also been developed to simultaneously detect DNA and RNA viruses in shrimps. In this regard, a study conducted in 2021 developed a dual priming oligonucleotide (DPO)- based multiplex PCR system which is cost effective. This multiplex PCR kit is time-saving, as results can be obtained within a day (See et al., 2021).

Another technology that has been implemented widely in this field is biosensing. Biosensors detect pathogens or diagnose diseases based on the conversion of biological responses generated during protein or nucleic acid interactions into electrical signals (Santos et al., 2020). This method can be used to diagnose and detect pathogens in aquaculture systems as well. Graphene oxide immobilized with methylene blue was used to develop an electrochemical immunosensor to successfully detect the presence of WSSV in penaeid shrimp (Natarajan et al., 2017). This method allows for the electrochemical immunosensing of the virus in the tissues of shrimps. In 2018, DNA Schottky diodes were used to derive electronic properties of DNA to develop a biosensor which can detect both bacterial and viral pathogens in penaeid shrimp samples (Rizan et al., 2018). DNA-based biosensors can become a significant tool in the diagnosis of microbial pathogens in shrimp farms in the field.

Nanotechnology is another advanced technology that has been applied in multiple areas of scientific research. However, the application of nanotechnology in the aquaculture industry is still novel. In Thailand, a rapid and efficient immunochromatographic test strip was developed using a monoclonal antibody W29 coupled with colloidal gold nanoparticles, to detect WSSV in *M. rosenbergii* (Sithigorngul et al., 2006). In addition, gold nanoparticles were also used to develop a Surface Plasmon Resonance (SPR^2^) device for the detection of WSSV (Lei et al., 2008).

ELISA is the currently used viral pathogen detection method in shrimp aquaculture (Ganjoor, 2015), which can be further improved to detect microbial diseases. For instance, white tail disease in *M. rosenbergii* was detected via a sandwich enzyme-linked immunosorbent assay (S-ELISA). This method was based on unlabeled antibody and biotinylated antibody coatings to trap antigens. S-ELISA is a rapid, sensitive, and cost-effective method that can be used in epidemiological studies of shrimp diseases (Romestand and Bonami, 2003).

Alternative perspectives can also be taken in the development of effective diagnostic methods for shrimp pathogens. An example would be the possible development and utilization of a new type of pathogen detection approach involving Raman spectra analysis using deep learning methods (Yu et al., 2021).

### 3.2 Vaccination/immunostimulants and immune memory of shrimps

Conventional vaccines consist of inactive pathogen-derived molecules such as antigens, that can trigger a host memory-based immune response to fight certain pathogens. This mechanism is known as adaptive immunity and is absent in invertebrates such as penaeid shrimps (Flegel, 2019). Hence, it has been a challenge to develop vaccines against viral and bacterial diseases of shrimps. It is significant to comprehensively understand the immune system of penaeid shrimps to develop effective control strategies. Immunological studies of shrimps have discovered a phenomenon that is similar to acquired immunity observed in vertebrates. In shrimps, the innate immune system can form immunological memory, and this is known as ‘trained innate immunity’ or ‘immune memory’ (Netea et al., 2011; Quintin et al., 2014; Boraschi and Italiani, 2018).

Multiple studies have reported the activation of innate immune responses in shrimps following an infection or the administration of immunization, which decreased the risk of getting infected by the same virus or reduced the spread of infection. Penaeid shrimps have exhibited resistance to experimentally induced WSSV for 4 months (Venegas et al., 2000; Wu et al., 2002). Moreover, when formalin inactivated WSSV or envelope proteins of recombinant WSSV were administered to shrimps, it also induced resistance (Namikoshi et al., 2004; Vaseeharan et al., 2006; Witteveldt et al., 2006; Ha et al., 2008). Protective levels of 50-90% have been observed with the administration WSSV envelope proteins, however, the duration of immunity depends on the formulation (Wu and Zhang, 2007; Wu et al., 2008). A recent study has suggested an epitope that could be used in the development of a vaccine against WSSV as well (Momtaz et al., 2019). The precise mechanism of action of DNA vaccines in shrimps is not completely understood, hence further research is required in this area. The use of immunostimulants is preferred as natural derivatives that range from bacteria, fungi, plants, animals, phytochemicals, and hormones, serving as primers for a chain of events in the PAMPs defense mechanism to clear the pathogen (Lee et al., 2020; Kumar et al., 2023). It is proposed that immunostimulants are a better chemotherapeutic than vaccines, as immune priming can be administered at the larval stage. Hence, disease intervention at the larval stage is a crucial step in ensuring higher survival rate in shrimp aquaculture. This strategy will pave way for reducing antibiotic use in shrimp aquaculture (Kumar et al., 2023). It is proposed that immunostimulants may be more effective than vaccines as a chemotherapeutic strategy in shrimp aquaculture. Applications of vaccines range from traditional killed/inactivated and live attenuated vaccines to new generation ones, including recombinant, synthetic peptides, mucosal and DNA, subunit, nanoparticle-based and plant-based edible vaccines, reverse vaccinology, and monovalent and polyvalent vaccines (Mondal and Thomas, 2022). In shrimp vaccines, the most promising vaccine that has been tested is against vibriosis, as discussed in a recent review by Amatul-Samahah et al., 2020. The aim of a good vaccine is based on a few criteria: 1) safe for shrimp and humans, 2) long lasting protection of the pathogens throughout the entire production cycle, 3) cost-effective and sustainable 4) trans-generational potential that can be passed to progeny, and 5) applicability across different shrimp species (Sivasankar et al., 2017; Kumar et al., 2018).

### 3.3 Phage therapy

Antibiotics are frequently used in shrimp aquaculture to treat bacterial outbreaks; however, there have been concerns regarding development of antibiotic-resistance in bacterial cultures and environmental pollution. One of the most common bacterial diseases reported in penaeid shrimp farm is vibriosis. Phage therapy is used to control and prevent bacterial infections in aquaculture systems through the use of lytic phages, which are viruses that infect bacteria, called bacteriophages (Flegel et al., 2008; Kalatzis et al., 2018). This technology has been promising (Defoirdt et al., 2011; Oliveira et al., 2012; Richards, 2014) ever since it was first applied in Japan to control *Lactococcus garvieae* communities (Nakai et al., 1999).

Over the last decade, phage therapy has been applied to treat diseases caused by various species of bacteria. In *L. vannamei*, Lomelí-Ortega and Martínez-Díaz (2014) administered A3S and Vp1 bacteriophages after 6 hours after a vibriosis infection caused by *V. parahaemolyticus* and reported a reduced mortality rate (Lomelí-Ortega and Martínez-Díaz, 2014). A similar study on the same species induced experimental phage therapy against *V. parahaemolyticus* causing AHPND and reported an increase in survival rates (Jun et al., 2018). This technology has also been applied against *V. alginolyticus* in *Apostichopus japonicus* (Zhang et al., 2015) and *V. harveyi* in *P. monodon* (Stalin and Srinivasan, 2017). However, there is a limitation of field studies involving this technology. Abiotic factors such as the quality of water and the composition of organic matter in the culture or pond, may influence the efficiency of phage therapy. Hence, further studies are required to establish models that determine the various external factors impacting the efficiency of phage therapy. Moreover, it is significant to conduct more studies to determine the optimal dosage and administration schedule of this treatment (Santos et al., 2020). Phage therapy has the potential to be a biologically safe, commercial, and environmentally friendly alternative to antibiotic treatment of bacterial diseases in penaeid shrimp aquaculture.

### 3.4 Quorum sensing (QS) to control virulence of bacteria

The development of antibiotic-resistance in bacterial species such as *Vibrio* is a major concern. There have been reports of antimicrobial-resistant (AMR) genes being transferred to human pathogens by *Vibrio* sp. Hence, it is imperative to develop methods to control AMR bacterial species in aquaculture systems (Bramhachari et al., 2019). QS is a bacterial mechanism that allows cell-to-cell communication and regulation of gene expression in response to cell density (Pawar and Lahiri, 2018). The production of virulence factors in bacteria is controlled by this mechanism. Consequently, QS has been extensively studied in disciplines such as medicine, environmental science, and technology. Recently, QS has been applied in the regulation of bacterial virulence and infections caused by *Vibrio* species in aquaculture (De Decker et al., 2013; Benitez and Silva, 2016). In this regard, QS was used to reduce the mortality associated with *V. campbelli* infection in brine shrimp larvae and *M. rosenbergii* (Pande et al., 2013). These studies suggest that QS system may be a potential target to develop treatments against shrimp infections caused by *Vibrio* spp.

### 3.5 RNA interference

RNA interference (RNAi) is a post-transcriptional process wherein dsRNA is introduced into cells to trigger the silencing of specific genes. When dsRNA is delivered into cells, it, along with proteins including Argonaute, forms the RNA-induced silencing complex (RISC). The RISC then targets, identifies, and degrades specific messenger RNA (mRNA) sequences, inhibiting translation of subsequent proteins (Agrawal et al., 2003). RNAi has potential applications in the disease management of penaeid shrimps (Walker and Mohan, 2009). This mechanism was first applied in shrimps against WSSV in the US (Robalino et al., 2004, 2005). Concurrently, RNAi was reported effective in protecting shrimps against YHV and TSV. Moreover, WSSV replication was inhibited in *M. japonicus* via the introduction of inactive bacteria expressing vp28-siRNA construct (Yodmuang et al., 2006; Xu et al., 2007; Zhu and Zhang, 2011). Furthermore, via the introduction of non-specific dsRNA constructs, the mortality rates of *M. japonicus* decreased when infected with WSSV (Maralit et al., 2015).

RNAi can also be used to study the function of proteins that are involved in the immune system of shrimps. Furthermore, the addition of dsRNA as feed additives in shrimp farms and breeding programs, may have the potential to protect against pathogens and generate pathogen-free progeny (Itsathitphaisarn et al., 2017). Limitations of RNAi in shrimp disease management include inadequate assessments of the long-term durability of the immunity induced by this strategy (Walker and Mohan, 2009). Moreover, the application of RNAi is still novel in this industry, as majority of the studies are conducted in laboratories (Loy et al., 2013). Hence, it is imperative to develop economic and practical techniques for RNAi application in shrimp disease management on a commercial level.

### 3.6 The future of NGS analysis in shrimp aquaculture

NGS platforms offer rapid, high throughput and large amounts of sequencing data. Third-generation sequencers such as PacBio and Oxford Nanopore, allow longer read lengths of ~20kb without the requirement for assembly processing (Amarasinghe et al., 2020). One such example is the complete transcriptome of *L. vannamei* by long-read sequencing (Zeng et al., 2018). Currently, the only reported complete shrimp genome is of *L. vannamei* (Zhang et al., 2019), while there are reports on the full-length transcriptomes of *P. indicus* and *P. monodon* (Huerlimann et al., 2018; Katneni et al., 2020). The development of bioinformatics offers novel advanced software for the assembly of genomes and genome annotation. Hence, with the advancement of NGS technologies and algorithms, future research can focus on generating reference genomes and transcriptomes for other commercially significant shrimp species. These sequencing data create valuable genomic resources that can be utilized in areas such as MAS in breeding programs, enhancing the sustainability of shrimp aquaculture (Shekhar et al., 2021).

Future research on whole genome sequencing of shrimp can focus on identifying genome-wide variations in wild and pondreared shrimps from different regions of the world. Moreover, the development of experimental and statistical methods is required to identify variations in genes and alleles that lead to desirable traits. In addition, it is crucial to enhance the integration of molecular and genomics tools in both academic and industrial settings. Furthermore, epigenomics studies help in understanding the influence of abiotic factors on shrimp phenotypes, thereby increasing the sustainability and productivity (Metzger and Schulte, 2016; Wan et al., 2016; Yue and Wang, 2017).

## 4 Epilogue

Since the outbreaks of shrimp diseases emerged in the 1980s, control measures have been implemented to prevent and reduce the spread of the diseases, primarily bacterial and viral infections. These measures mostly involved onsite biosecurity, the use of probiotics, and the development of SPF and SPR^1^ stocks. Multiple molecular diagnostic tools, including PCR-based methods, monoclonal antibody assays, and lateral flow chromatographic assay test strips, were developed to detect and diagnose various pathogens. Crucially, some of these methods enabled farmers to detect and diagnose pathogenic microorganisms directly at shrimp farms, significantly enhancing disease management and prevention efforts. However, a significant concern persists regarding the lack of effective strategies to prevent the emergence of new or previously controlled infections and to treat them. Due to the absence of an adaptive immune system, conventional disease prevention measures like vaccination are ineffective in shrimp. Consequently, numerous studies have concentrated on elucidating the underlying mechanisms of shrimp immune responses, potentially advancing our understanding of immunomodulation. Over the past few years, improved pathogen detection methods, including the application of NGS platforms, nanotechnology, CRISPR-Cas9, and DNA biosensors, have gained traction. Additionally, novel technologies with the potential to treat shrimp diseases, such as the development of vaccines, phage therapy, quorum sensing to control bacterial virulence, and gene silencing using RNA interference, show great promise. However, additional field studies are necessary to evaluate the effectiveness of these strategies in treating or preventing the emergence of diseases in shrimp aquaculture systems. This research is crucial to ensure that these systems become profitable and sustainable for farmers, while also being safe for consumers. The next frontier research should be directed towards the development of culture-free diagnostics. This approach would ideally require transdisciplinary methodologies such as those harnessing physiochemical biosensors.

## Figures and Tables

**TABLE 1:** Viral shrimp diseases that are listed by OIE (Lightner et al., 2012) and updated by Lee D et al., 2022.

Viral Disease name	Name of the pathogen	Genome	Family	Stage	Symptoms
Taura syndrome (TS)	Taura syndrome virus (TSV)	ssRNA	Dicistroviridae	Late post larval to early juvenile stages, between 15–40 days, but it can also induce serious diseases in both sub-adult and adult *P. vannamei*	Chromatophore expansion) and irregular black (melanization) spots under the cuticle layer, in addition to anorexia, an erratic swimming behavior, lethargy, soft cuticles, anorexia, flaccid bodies and opaque musculature
White spot disease (WSD)	White spot syndrome virus (WSSV)	dsDNA	Nimaviridae	During all stages of development, from egg to adult, species are vulnerable to WSSV	anorexia, lethargy, abnormal behavior (decreased swimming ability, disorientation and swimming on one side), red discoloration of the body surface (uropods, telson, pereiopods, and pleopods), swelling of branchiostegites, a loosening of the cuticle, enlargement and yellowish discoloration of the hepatopancreas, thinning and delayed clotting of hemolymph characteristic white spots with a diameter of 1–2 mm (or 0.5–3.0 mm) on the carapace, appendages, and internal surfaces during disease progression
Yellow head disease (YHD)	Yellow head virus (YHV) & Gill-associated virus (GAV)	ssRNA	Roniviridae	Nursery population at approximately 60 days post stocking	yellow coloration of the cephalothorax and gills,
Infectious hypodermal and hematopoietic necrosis (IHHN)	Infectious hypodermal and hematopoietic necrosis virus (IHHNV)	ssDNA	Parvoviridae	Nursery population at approximately 60 days post stocking	cuticular deformities of the rostrum, antennae, thoracic and abdominal areas
Infectious myonecrosis (IMN)	Infectious myonecrosis virus (IMNV)	dsRNA	Totiviridae	Nursery population at approximately 60 days post stocking	focal to extensive white necrotic areas in the striated muscle, especially the distal abdominal segments and tail fan, slow mortality that persists during the culture period (cumulative mortality reaching up to 70%)
Necrotizing Hepatopancreatitis (NHP)	Hepatobacterium penaei	Rickettsia-like bacteria	Holosporaceae	Nursery population at approximately 60 days post stocking	Necrotic regions in striated muscles that appears red (Nur’aini, 2009). In the acute phase of IMN, coagulative muscle necrosis can be observed with oedema of infected muscles, leading to fluid retention between muscle fibers and infiltration of hemocytes. The infection may further lead to hypertrophy due to lymphoid organ spheroids (LOSs) that generally appear in heart, gills, ventral nerve cord, and close to antennal gland tubules.

The shrimp morphology pictures and its symptoms infected with the virus is updated by Lee D et al, (2022).
